# Effect of Modifiable Lifestyle Factors on Biological Aging

**DOI:** 10.14283/jarlife.2024.13

**Published:** 2024-06-05

**Authors:** W.-H. Lu

**Affiliations:** 1. IHU HealthAge, Toulouse, France;; 2. Institute on Aging, Toulouse University Hospital (CHU Toulouse), Toulouse, France.

**Keywords:** Healthy aging, healthspan, biomarker of aging, epigenetic age, age acceleration

## Abstract

Biological age is a concept that uses bio-physiological parameters to account for individual heterogeneity in the biological processes driving aging and aims to enhance the prediction of age-related clinical conditions compared to chronological age. Although engaging in healthy lifestyle behaviors has been linked to a lower mortality risk and a reduced incidence of chronic diseases, it remains unclear to what extent these health benefits result from slowing the pace of the biological aging process. This short review summarized how modifiable lifestyle factors — including diet, physical activity, smoking, alcohol consumption, and the aggregate of multiple healthy behaviors — were associated with established estimates of biological age based on clinical or cellular/molecular markers, including Klemera-Doubal Method biological age, homeostatic dysregulation, phenotypic age, DNA methylation age, and telomere length. In brief, the available studies tend to show a consistent association of lifestyle factors with physiological measures of biological age, while findings regarding molecular-based metrics vary. The limited evidence highlights the need for further research in this field, particularly with a life-course approach.

**A**ging is the time-related deterioration that occurs in an organism at all levels, from the molecular and cellular to the physiological and functional, ultimately increasing vulnerability to death (1). For decades, scientists and clinicians have observed that chronological age, representing the time since birth, is a significant predictor of various age-related health conditions; however, it may not accurately describe how an organism functions, especially in the later life stages (2, 3). Biological age seeks to quantify the bio-physiological processes driving aging. Generally, biomarkers or clinical metrics designed to forecast the remaining lifespan and healthspan (the absence of disability) are considered indicators of biological age (4, 5). The disparity between predicted biological and chronological age is defined as age acceleration, and the positive age acceleration implies that individuals may undergo age-related decline faster than their peers (6, 7).

Engaging in healthy lifestyle behaviors has been linked to a lower mortality risk (8) and decreased incidence of a myriad of medical conditions, including cardiovascular diseases (9), metabolic syndrome (10), cancer (11), neurodegenerative and psychiatric disorders (12), and geriatric syndromes (13, 14). Although the mechanisms connecting lifestyle factors to extended lifespan/ healthspan are not fully understood, it is plausible that the health benefits result, at least partly, from slowing down the biological aging process. This short review explored how modifiable lifestyle factors, such as diet, physical activity, smoking, and alcohol consumption, are associated with biological aging.

## Biological age estimation

Articles published in English were searched from the Pubmed database for this review. Several papers investigating the relationship between modifiable lifestyle factors and biological age using various measures from physiological to molecular scales were identified. Only research that quantified biological age using validated algorithms, epigenetic clocks, or telomere length were included (see Table 1).

**Table 1.** Biological age estimation methods used in prior studies on modifiable lifestyle factors

Name	Methods
Klemera-Doubal Method biological age (KDM-BA) (15)	KDM-BA is derived from a series of regressions between specific biomarkers and chronological age. It represents the age at which the average physiology in a reference sample aligns with that of the subject (16).
Homeostatic dysregulation (HD) (17)	HD indicates the degree to which a subject’s physiological measurements deviate from reference health values, using the Mahalanobis distance based on a set of biomarkers from multiple systems.
Phenotypic age (PA) (18)	PA integrates blood-chemistry biomarkers and chronological age to estimate the 10-year mortality risk, which is then converted into units of years. It was employed to train the composite epigenetic clock known as DNAm PhenoAge.
Multidimensional aging measure (MDAge) (19)	MDAge is calculated based on a linear combination of chronological age and 13 clinical chemistry biomarkers that were selected using random forest algorithm.
DNA methylation age (DNAmAge)	DNAmAge, also known as epigenetic age, is an estimated age based on the DNA methylation pattern across specific CpG sites (20). DNAmAge clocks may be trained either on chronological age (such as the Hannum and Horvath clocks) or on age-related phenotypes and/or biomarkers (such as PhenoAge and GrimAge).
Telomere length	Telomeres are repetitive DNA sequences located at the ends of chromosomes to protect their integrity from degradation during mitosis. Telomere shortening is correlated with increased chronological age (21) and may contributed to the onset of aging related pathologies (22).
Frailty index (FI)	Frailty is a vulnerable status resulting from the multi-level deterioration across physiological systems and associated with higher risk of mortality and adverse events. FI is a measure of frailty calculated by determining the proportion of clinical deficits present to the total (23, 24).

## Diet and biological aging

An unhealthy diet may accelerate biological aging due to its inflammatory and oxidative stress potentials. The cross-sectional study conducted by Wang and colleagues, which involved 8,839 participants from the National Health and Nutrition Examination Survey (NHANES) of the United States, showed a consistent association of consuming foods with higher Dietary Inflammatory Index (DII) and Dietary Oxidative Balance Score (DOBS) with accelerated biological aging. In this work, biological age was assessed through clinical biomarkers using established algorithms, including Klemera-Doubal Method biological age (KDM-BA), homeostatic dysregulation (HD), and phenotypic age (PA) (25). Another study of 10,191 Taiwanese aged ≥50 revealed that adopting a diet rich in plant foods was associated with a reduced likelihood of experiencing an acceleration in the multidimensional aging measure (MDAge) over 8 years, composed of selected clinical chemistry biomarkers (26). Kresovich et al.’s cross-sectional study demonstrated the beneficial impact of healthy eating approaches, including the diet designed for hypertension management and the Mediterranean diet (MED), on DNA methylation age (DNAmAge) acceleration among non-Hispanic white women (the Sister study); the most significant associations were observed in acceleration in PhenoAge and GrimAge (27). Conversely, an 18-month randomized controlled trial (RCT) in 294 adults with obesity or dyslipidemia observed no significant differences in the change of epigenetic ages between three dietary interventions, which included providing guidelines to promote a healthy diet and implementing a calorie-restricted MED and a plant-rich MED, respectively (28). In summary, the observational studies suggest that a healthy diet may decelerate biological aging, while further evidence is required to determine whether different dietary strategies are superior.

## Physical activity and biological aging

Several non-interventional studies had reported that higher physical activity levels or lower sedentary time were associated with slower epigenetic aging (29–31). However, the association may be partially attributed to body mass index (BMI) and other confounders, with the associations tending to attenuate or disappear after adjusting for those confounders (29, 30). Further insights from Fox and colleagues revealed that cardiovascular health and immune function mediated the effect of physical activity on DNAm GrimAge acceleration (31). Physical activity also showed a favorable impact on telomere attrition. In their study recruiting 284,479 participants from the UK Biobank, Zhu et al. discovered that physical activities during leisure time, housework, and public transportation were associated with reduced leukocyte telomere length (LTL) deviation, which referred to the difference between genetically determined and observed LTL. Notably, engaging in job-related activities was linked to a greater LTL deviation (32). In short, engaging in physical activities outside of work could slow down the rate of biological age acceleration, as measured by cellular markers.

## Smoking, alcohol consumption, and biological aging

As calculated by KDM-BA and PA, individuals who smoked and drank alcohol had a greater age acceleration than those who reported as non-smokers/non-drinkers, with evidence from 94,433 adults aged 30 to 70 in Taiwan (33). This finding is supported by a study investigating epigenetic age among 2,316 women from the Sister study, which indicated that both lifetime and recent alcohol consumption were associated with DNAm GrimAge acceleration (34). Furthermore, smoking and alcohol consumption were cross-sectionally associated with acceleration in several DNAmAge clocks in the GENOA study composed of 1,100 African Americans; however, only current smokers showed a significant association with increased PhenoAge acceleration over time (35). To summarize, tobacco and alcohol consumption have been correlated with accelerated biological aging, as demonstrated by cross-sectional studies, but longitudinal evidence supporting these associations remains insufficient.

## Multiple lifestyle factors and biological aging

The effect of engaging in multiple healthy behaviors on deceleration in biological aging had also been evaluated, including nonsmoking, less alcohol consumption, daily fruit and vegetable intake, being physically active or regular exercise, good sleep habits, and maintaining normal BMI and waist-to-hip ratio (36–38). Overall, adherence to more health-promoting factors was associated with slower biological aging, either assessed via the phenotypic measure (frailty index) (36) or clinical biomarkers (KDM-BA and PA) (37, 38).

Despite limited sample sizes, data from RCTs suggested that lifestyle interventions may modify biological age. The pilot trial of Fitzgerald et al. performed an 8-week treatment program about diet, dietary supplements, sleep, exercise, and stress management for 43 men aged 50 to 72 without chronic diseases. Compared to the controls, the lifestyle intervention was associated with a decrease in Horvath DNAmAge of 3.23 years. Moreover, in the intervention group, Horvath DNAmAge decreased by an average of 1.96 years by the end of the program (not reaching statistical significance) (39). In a secondary analysis of a 24-month RCT that enrolled 219 healthy post-menopausal women, participants who received the healthy-dietary intervention had a lower GrimAge acceleration than their no-intervention counterparts. On the other hand, the physical activity intervention reduced the epigenetic mutation load (40), which reflects the age-related dysfunction of the epigenetic maintenance system (41). Finally, a secondary analysis of an RCT involving 93 obese older adults observed that a 12-month calorie-restricted diet, whether combined with exercise or not, was associated with decreased biological age as per three different algorithms. In contrast, the exercise intervention alone did not significantly alter biological age over time and showed no difference from controls (42). To sum up, multiple healthy behaviors may collectively slow biological aging.

## Perspectives on the way forward

**Figure 1.**
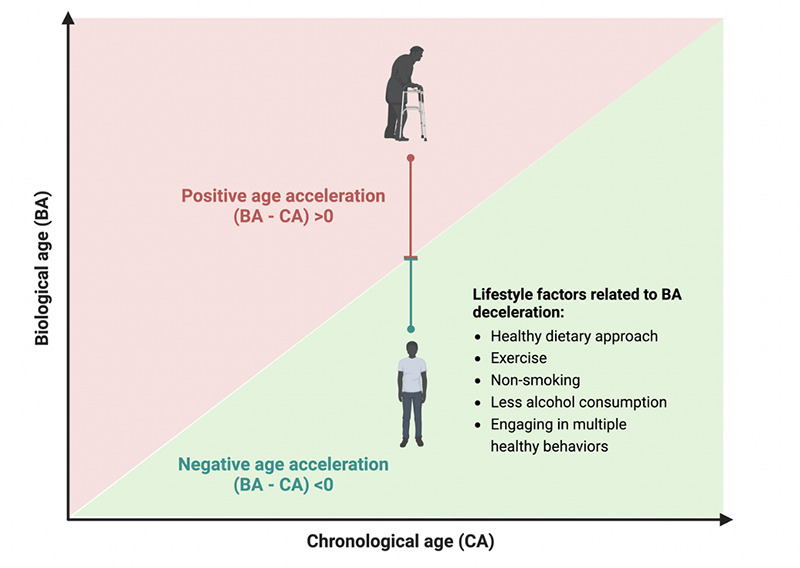
Lifestyle factors showed an association with the deceleration of biological age suggested in prior studies

Note: This figure was created with BioRender.com.

Individuals who engage in a healthy lifestyle may exhibit a slower pace of biological aging, as their DNA methylation profile and physiological biomarkers are in a healthier state that typically indicates lower risks of mortality and age-related diseases (Figure 1). However, most studies linking lifestyle factors and biological aging are cross-sectional designs, making it difficult to establish causation. Furthermore, it is worth noting that previous research investigating lifestyle factors and biological aging was commonly obtained from specific US cohorts, such as NHANES and the Sister study, probably due to the difficulty of having both biological age measures and comprehensive lifestyle data in other large cohorts. More evidence derived from diverse populations needs to be included. The impact of lifestyle factors on biological aging warrants investigation using a life-course approach. It is possible that alterations in biological mechanisms become evident only if these behaviors start at a younger age or are consistently adopted in the long term. Due to the difficulty of following individuals throughout their lifespans, initiatives such as the INSPIRE project (43) are crucial for contributing to this topic, as they enable the following of a large age range over a relatively long period. Finally, larger-sample RCTs are needed to validate observed effects.

Several measurement issues of biological age also remain in the field. For example, even if the same biological age algorithm is used, employing different biomarker selection strategies may result in the diverse compositions of the biomarkers and, thus, different performance of estimated biological age (44). Similarly, the lack of standardization in biomarker formulations and study design/performing procedures can lead to heterogeneous results when examining aging biomarkers across cohorts (45). Measuring and parameterizing biological age will continue to pose challenges in future observational studies or RCTs on lifestyle factors. Notably, recently proposed guidelines for validating biomarkers of aging offer a solution to harmonize future cross-population studies, which provide several recommendations for investigating omics-based aging biomarkers at different stages, from data maintenance and biomarker development to external validation (45). Lastly, using digital markers collected by wearable sensors for measuring biological age is a promising field that requires further exploration. A previous study showed that biological age acceleration estimated from step count data could distinguish morbidity and smoking status as effectively as blood-based markers (46). However, it remains to be investigated how this digital biomarker-based measure of biological age can help reveal the impact of modifiable lifestyle factors.

Some interventions demonstrated symptom-relieved effects without significantly altering the underlying pathology (47); the same question could be posed regarding the biological influence of lifestyle behaviors discussed in this article. The available evidence tends to show a consistent association of lifestyle factors with physiological measures of biological age, while findings regarding molecular-based metrics (especially epigenetic clocks) vary. This suggests that lifestyle factors have a greater impact on physiological health, reflecting the overall accumulation of cellular and molecular damage, rather than targeting a specific aging mechanism. Future research comparing multiple biological aging measures derived from different levels of organization within the body (physiological, cellular, and molecular) can provide insight into the topic. In addition, given that health-promoting factors have been shown to modify the association between disease pathologies and phenotypic outcomes — such as the role of physical activity in neurodegenerative diseases (48) — it is important to investigate whether these modifying effects result from the decelerated biological aging and the enhanced biological resilience.
